# Patients’ and professionals’ preferences in terms of the attributes of home enteral nutrition products in Spain. A discrete choice experiment

**DOI:** 10.1038/s41430-017-0023-8

**Published:** 2017-12-20

**Authors:** Gabriel Olveira, Miguel Ángel Martínez-Olmos, Belén Fernández de Bobadilla, Mercedes Ferrer, Nuria Virgili, Belén Vega, Mercedes Blanco, Miquel Layola, Luis Lizán, Irmina Gozalbo

**Affiliations:** 1 UGC Endocrinología y Nutrición. Hospital Regional Universitario de Málaga/Universidad de Málaga, IBIMA, Málaga Spain; 2 Hospital Universitario de Santiago, A Coruña, Spain; 3 Hospital General Universitario de Ciudad Real, Ciudad Real, Spain; 4 Hospital Universitario Clínico Virgen de la Arrixaca, Murcia, Spain; 5 Hospital Universitario de Bellvitge, Barcelona, Spain; 6 Hospital Universitario Ramón y Cajal, Madrid, Spain; 7 Nestlé Health Science, Barcelona, Spain; 8 Outcomes’10, Castellón, Spain

## Abstract

**Background/objectives:**

To elicit and compare preferences in terms of the attributes of home enteral nutrition (HEN) among patients and physicians, using a discrete choice experiment (DCE).

**Subjects/methods:**

A DCE comprising eight choice scenarios, with six HEN attributes (tolerability, adaptation to comorbidities, nutrition and calories, handling, connections and information; two levels each) was designed. The Relative Importance (RI) for patients and physicians of each attribute was estimated. Sociodemographic and clinical variables, as well as additional questions (*n* = 8) were compiled to analyze possible explanatory variables and other preferences.

**Results:**

A total of 148 HEN patients (71 needing caregivers to answer on their behalf) and 114 physicians completed the DCE. The most important attributes for patients were *adaptation to comorbidities* (33% RI), *tolerability* (33% RI), and *nutrition and calories* (26% RI). Significantly, younger patients had stronger preferences for *tolerability* whereas elderly ones (≥75 years) were more concerned about *handling*. In comparison, physicians gave a higher RI to *tolerability*, and *nutrition and calories* compared to patients (*p* = 0.002). Overall, a higher percentage of physicians answered that HEN characteristics such as easy-handling bags (85.1 vs. 64.9%; *p* = 0.001), container material (69.3 vs. 57.1%; *p* = 0.003) or reusable containers (79.8 vs. 70.3%; *p* = 0.01) were “important” or “very important” compared to patients.

**Conclusions:**

Our findings showed that although patients and physicians have a similar perception about the relevance of different HEN attributes, the relative weight given to each one varies between them. Therefore, both points of view should be considered when choosing a HEN product in order to improve patients’ satisfaction and clinical outcomes.

## Introduction

The modality of Home Enteral Nutrition (HEN) responds to the needs of individuals who are functionally able to live at home, but require assistance to maintain their nutritional status due to their chronic conditions [1, 2]. The use of HEN has increased considerably over the last decades [3], taking advantage of the new technologies available, and helping to reduce hospitalizations [4–6]. Nevertheless, HEN’s real prevalence is difficult to determine because of differences between country legislations and registries, as well as the different types of HEN considered in each evaluation [5, 7]. In Spain, a prevalence of 221 patients receiving enteral tube feeding per million inhabitants was estimated in 2007 [8] according to the defined daily dose methodology, whereas the latest HEN (voluntary) registry showed a rate of prevalence of 80.58/10 [6] inhabitants, in 2014, and 90.51/10 [6] inhabitants, in 2015 [9].

HEN has improved patients’ nutritional status, reducing the number of admissions and hospital stays, and hence decreasing costs related to long hospitalization periods [2, 10, 11]. HEN has also allowed patients to increase family conciliation and health-related quality of life (HRQoL) [9, 12]. However, despite the numerous benefits associated with this therapy, some studies have revealed a high frequency of mechanical or gastrointestinal complications [13–15]. In a Spanish prospective study [14], 42% of patients receiving HEN had complications, the most frequent being mechanical (extraction) (15%), followed by digestive (constipation (13%), vomiting (12%), and diarrhea (10%)).

In light of the widespread need for HEN, the possible presence of complications related to this treatment, and the variety of products available to meet patients’ different needs [2], a better understanding of patients’ and physicians’ preferences for treatment characteristics might help prescribers make more accurate choices. This would make patients feel more comfortable and involved in their treatment, thus giving rise to better adherence to treatment. This involvement ensures enhanced treatment efficacy [16], patients’ HRQoL [17] and reduced medical costs [18], as it means that patients take the right doses at the right time as per medical or health advice [19].

Unfortunately, there is little knowledge about patients’ and healthcare professionals’ priorities and needs with regard to HEN.

The discrete choice experiment (DCE) [20] is a relatively new methodology, whose aim is to elicit preferences for products or interventions, which has experienced a growing use over the last few years in healthcare [21, 22]. It is grounded in the principle that individuals make rational choices based on the product characteristics, as well as on their own needs and priorities. In this way, DCEs enable preferences among the main stakeholders to be measured, increasing the information available to decision-makers in terms of their stated preferences and needs [23].

The aim of this study is to assess and compare preferences for different characteristics of HEN via tube feeding among patients and physicians, using a DCE.

## Materials and methods

### Design of the experiment

The study was an observational, multicentre, exploratory study performed in real clinical practice in Spain.

This DCE was applied according to the International Society for Pharmacoeconomics and Outcomes Research (ISPOR) good practices recommendations for conjoint analysis in healthcare [24].

### Discrete choice experiment

#### General description

DCEs are regularly used in health economics to elicit preferences for healthcare interventions and products, as they allow identifying how much responders value each of their defining characteristics [25]. In DCEs, individuals repeatedly choose between two or more hypothetical treatment alternatives described by attributes (defining characteristics) and their corresponding levels (different possible values those attributes can take) [26]. The choices made are analysed to determine and measure which are the most preferred attributes and levels.

#### Attributes and level selection

A literature review was conducted using key terms (Supplementary Table 1) to search the international databases of MedLine/PubMed, Cochrane Library and ISI Web Of Knowledge, reviewing those studies and assessing the preferences for HEN attributes. A total of six previous studies related to HEN preferences were identified [26–31], leading to 12 attributes with 2–3 levels each.

Two focus groups were invited to evaluate and choose the definitive attributes and levels. Six physicians with proven experience in HEN prescription formed the scientific committee that would recruit the rest of participants. The first focus group (*n* = 6) was comprised of this scientific committee, while the second group included patients (identified by the former) receiving HEN (*n* = 6) and caregivers (*n* = 5).

Both groups commented which attributes and levels they considered relevant when choosing a HEN product. Finally, 6 HEN attributes, with 2 levels each, were selected to be included in the DCE: “tolerability”, “adaptation to comorbidities”, “nutrition and calories”, “container characteristics” (“handling” from now on), “connections between tube and administration system” (“connections” from now on), and “information” (meaning the information available on the container) (Table 1).

**Table 1:** Final attributes and levels used in the discrete choice experiment

Attributes	Levels
Tolerability	Easily tolerable
	Hardly tolerable
Adaptation to comorbidities	Adaptable to other comorbidities present
	Not adaptable to other comorbidities present
Nutrients and calories	Provides the nutrients and calories needed by the patient
	Does not provide the nutrients and calories needed by the patient
Container characteristics	Its characteristics make package handling easier
	Its characteristics make package handling harder
Connections between the container and the feeding tube	Product connections are easy to perform
	Product connections are hard to perform
Information	The container includes information about the nutrient composition and branding
	The container does not include information about the nutrient composition and branding

#### Experimental design

The support CEs package for R3.2.2 [32] was used to generate the DCE design. This design was done in agreement with the recommendation of the ISPOR good practices for conjoint analysis in health [11] so that it was orthogonal (all attribute levels vary independently) and balanced (each attribute level occurs the same number of times). The fractional factorial analysis reduced the number of scenarios needed, while the mix-and-match algorithm [32] generated the pairs of choice.

A total of eight scenarios were created, each including two hypothetical HEN products defined by different levels of the six attributes considered (Supplementary Table 2). Supplementary Figure 1 presents an example of the choice set as included in the final questionnaire.

### Survey instrument

Two different questionnaires were generated, one for patients and their caregivers, and one for the physicians. The questionnaires contained the same DCE choice scenarios but included some sociodemographic and clinical variables in the case of patients and their caregivers, and other sociodemographic and professional variables in the case of physicians. These additional questions were introduced in order to explore whether they might be explanatory for the stated preferences.

The surveys were completed with a set of ad-hoc questions, which were the same for both groups. This self-administered questionnaire (5-Likert scale) was designed to assess the importance placed by patients and physicians on 8 alternative HEN features that were also found to be important by the focus groups, but not sufficiently important so as to be included in the DCE. The features considered were “easy-handling bag”, “container size”, “container weight”, “container material”, “allows oral and tube feeding”, “reusable container”, “duration of administration”, and “variety of flavors available”. Responders were asked to answer whether they found each of these HEN characteristics “unimportant”, “limitedly important”, “neutral”, “important” or “very important”.

### Study participants

#### Inclusion criteria

Patients aged > 18 years old currently receiving HEN via tube feeding or having received it during the previous year. Patients had to be monitored by professionals specialized in clinical nutrition within the Spanish NHS.

For patients who satisfied the inclusion criteria but were unable to answer by themselves, their caregivers (>18) were invited to answer on their behalf.

Specialized physicians from Spanish NHS hospitals and with vast experience in prescribing to and/or monitoring HEN patients were included.

All of the participants gave their written informed consent.

#### Study sample and data collection procedures

The sample size of both patients and physicians was estimated using Cochran’s formula [33], which estimates the minimum size needed for the sample to be representative of the total population, based on the population size and accepted error (*e* = 8%). The maximum variability criterion was applied with a confidence level of 95%. The sample size was increased by 5% in case some surveys had to be discarded.

The sample size for patients (*n* = 155) was based on the prevalence of HEN in Spain [8] and the general population [34]. The sample size for physicians (*n* = 100) was obtained from the total amount of specialized clinicians dedicated to clinical nutrition in Spain [35].

### Statistical analyses

A statistical descriptive analysis using SPSS Statistics 20.0 was conducted to describe participants’ demographic and clinical traits. Numerical data are given as median with 25 and 75 percentiles in brackets. The DCE was analyzed using the clogit function of the survival [36] package for R [37]. The conditional logit (clogit) estimates the partial utility values (PUV) associated with each attribute level, assuming that the choices made are based on the characteristics of the alternatives. The relative importance (RI) of each attribute was calculated as the quotient between the range of their PUVs and the sum of the PUV’ ranges of the whole set of attributes.

The mlogit function from the mlogit package [38] was used to evaluate the influence of the demographic (age, gender, location), clinical (diagnosis, time since HEN treatment, route and method of administration) and professional variables (gender, location, years of experience, patients per month, speciality). The multinomial logit (mlogit) assumes that individuals’ characteristics also influence the choice made. To assess differences between patients and physicians, two-sample z-tests were applied to the PUVs and the RIs [39–41] of both groups.

### Ethical consideration

This study followed the principles of the Declaration of Helsinki. It was developed to ensure compliance with Good Clinical Practices, in keeping with the principles of the Tripartite Harmonized ICH Guideline [22] (International Conference on Harmonization, ICH, 1996). The study protocol was submitted to the Spanish Agency of Medicines and Medical Devices (Agencia Española del Medicamento y Productos Sanitarios) for classification and to the Clinical Research Ethics Committee of each of the participating centres for approval.

## Results

### Participant demographics

#### Patients

The electronic questionnaire was completed by 148 patients who were included in the final data analysis. Of these, 71 needed caregivers to answer on their behalf. Median age was 67 years (54; 77.5), most of them were men (61.5%), and married or living with a partner (65.5%). Most of the patients had completed primary education (41.2%) and 63.5% were retired. Approximately 50% of patients reported diagnosis of neurological pathologies, 43.9% had some form of cancer while the rest of the cases presented different underlying conditions requiring HEN (6.1%). Patients had been receiving enteral nutrition for an average period of 9 months (3; 36), as their sole source of nutrition (87.8%) or as complementary feeding (12.2%); routes of administration were gastrostomy (67.6%), nasogastric/enteral tube (28.4%) or jejunostomy feeding tube (4%) (Table 2).

**Table 2:** Patients’ sociodemographic and clinical characteristics (*n* = 148)

Characteristic	% of patients or mean
Age, years, median (P_25_; P_75_)	67 (54;77.5)
Male, %	61.5
Marital status, %	
Married/living with partner	65.5
Widower/Widow	16.9
Single	13.5
Separated/divorced	4.1
Level of education, %	
Primary school	41.2
Secondary school/vocational training	10.1
University	10.8
Postgraduate	0
No studies	29.7
Employment status, %	
Retired	63.5
Employed, full or part-time	8.2
Unemployed/student	1.4
Long-term sick-leave/disabled	23
Other	4.1
Diagnosis, %	
Neurological pathologies	50
Cancer	44
Other	6
Time since HEN treatment, months, median (P_25_; P_75_ **)**	9 (3;36)
Route of administration (%)	
Gastrostomy	67.6
Nasogastric/enteral tube	28.4
Jejunostomy feeding tube	4
Method of administration (%)	
Gravity	61
Syringe	28
Infusion pump	12

#### Physicians

114 physicians completed the electronic questionnaire and were included in the final data analysis. Median age was 47 (38; 55), and most of them were women (64.9%; 95% CI:55.8–73.1). Most professionals were endocrinologists or nutritionists (87.7%; 95% CI:80.4–92.5), attending to more than 15 patients per month (61.4%; 95% CI:52.2–69.8). They had vast experience in clinical nutrition (>10 years) (67.3%; 95% CI:58.2–75.2).

### Participant preferences for HEN attributes

After conducting the analysis, the “information” attribute was found to be redundant (i.e., it could be explained by the rest of attributes) both for patients and physicians, while the “connections” attribute was found to be not significant (*p* = 0.734) for patients. In order to establish the importance assigned to each attribute, their RI was estimated. To exclude the effect of the non-significant attributes, a weighted RI was re-calculated by excluding “information” for both patients and physicians, and “connections” only for patients (Table 3 and

**Table 3:** Patients’ preferences for HEN characteristics

Attribute	Level	Partial utility	SE	P-value	RI	Weighted RI
Tolerability	Easy	2.088	0.214	<0.001	32.6%	32.9%
	Hard	−2.088				
Adaptation to comorbidities	Adaptable	2.095	0.240	<0.001	32.7%	32.9%
	Not adaptable	−2.095				
Nutrients and calories	Provides enough	1.642	0.129	<0.001	25.6%	25.8%
	Does not provide enough	−1.642				
Container characteristics	Easy to handle	0.527	0.108	<0.001	8.23%	8.29%
	Harder to handle	−0.527				
Connections	Easy to perform	0.527	0.153	0.734	0.81%	NA
	Hard to perform	−0.527				
Information	Includes	NA	0	NA	NA	NA
	Does not include	NA				

*RI* relative importance, *SE* standard errorand, *NA* not applicable

**Fig. 1:**
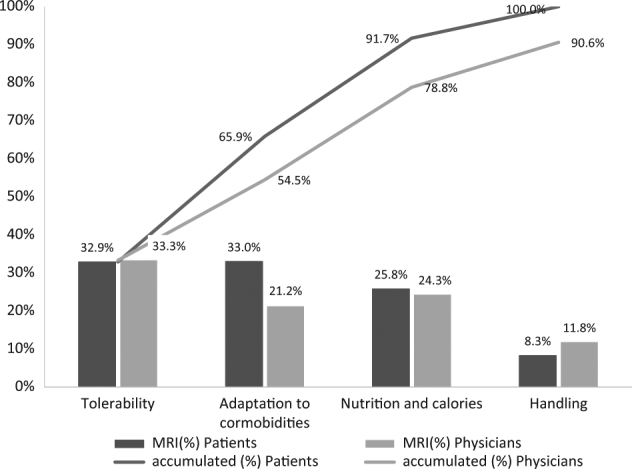
Patient’s and physicians’ preferences for HEN characteristics (*MRI* mean relative importance)

Table 4). As a result, “tolerability” was found to be the most important attribute (32.9–33.3% RI for patients and physicians, respectively) by both groups, tied with “adaptation to comorbidities” in the case of patients, and followed by “nutrients and calories” (25.8–24.3% RI). The least important attributes were “handling” (8.29–9.34% RI) and “connections” (9.34-0% RI) (Fig. 1).

**Table 4:** Physicians’ preferences for HEN characteristics

Attribute	Level	Partial utility	SE	P-value	RI	Weighted RI
Tolerability	Easy	3.32	0.036	<0.001	33.33%	33.33%
	Hard	−3.32				
Adaptation to comorbidities	Adaptable	2.11	0.121	<0.001	21.18%	21.18%
	Not adaptable	−2.11				
Nutrients and calories	Provides enough	2.42	0.089	<0.001	24.30%	24.30%
	Does not provide enough	−2.42				
Container characteristics	Easy to handle	1.18	0.308	<0.001	11.85%	11.85%
	Harder to handle	−1.18				
Connections	Easy to perform	0.93	0.395	<0.001	9.34%	9.34%
	Hard to perform	−0.93				
Information	Includes	NA	0	NA	NA	NA
	Does not include	NA				

*RI* relative importance, *SE* standard errorand, *NA* not applicable

#### Preference-defining factors

Once preferences were obtained, the sociodemographic, clinical and professional variables were analyzed in order to explore whether they could be considered explanatory for the preferences (Supplementary Table 3). Although physicians did not find any significant variables, patients’ preferences were found to be significantly influenced by age (*p* = 0.033). In this regard, patients < 75 years old would be more concerned about “tolerability” (33.2 vs. 29.9% RI) than older ones (≥75 years), whereas elderly patients would be more concerned about “handling” (9.0 vs. 7.5% RI) compared to subjects under 75-years old.

#### Comparison between patients’ and physicians’ preferences for HEN attributes

A Z-test was applied to compare the preferences obtained from the DCE analysis by patients and professionals. The analysis showed that physicians attached higher relative importance to “tolerability” (3.32 vs. 2.09; *p* = 0.002) and “nutrition and calories” (2.42 vs. 1.64; *p* = 0.007) compared to patients, while the other RIs did not significantly differ.

### Ad-hoc questionnaire

Answers to the ad-hoc questionnaire indicated that, overall, patients and physicians agreed upon the importance of HEN characteristics. However, a significantly higher proportion of physicians considered that certain container features such as “easy-handling bag” (85.1 vs. 64.9%; *p* = 0.001), “container material” (69.3 vs. 57.1%; *p* = 0.003) or “reusable container” (79.8 vs. 70.3%; *p* = 0.01) were important or very important, compared to patients. In addition, more physicians answered that they considered the “variety of flavors available” (72.8 vs. 54.7%) or the “duration of administration” (83.3 vs. 72.8%) to be important or very important, compared to patients.

## Discussion

To our knowledge, this is the first study to evaluate patients’ preferences for HEN characteristics using a Conjoint Analysis. We have observed that, although patients and physicians have a similar perception of the relevance of different HEN characteristics, physicians’ preferences are not fully in line with patients’ preferences.

HEN allows the administration of enteral formulas through the digestive tract, mainly by tube feeding, with the aim of avoiding or reducing malnutrition in patients who are cared for at home. Thus, HEN enables patients to remain within their socio-family environment while maintaining a very similar security profile and efficacy to that they would obtain in hospital. In this sense, enhancing patients’ adherence to HEN is thus of prime importance to extend their life and improve its quality.

Surprisingly, although meeting patients’ preferences is known to be a key factor that improves adherence, no similar studies have been published to date. Some studies can be found in the literature that address similar issues, related to flavors [31], palatability [29] or satisfaction [42]. However, these studies use questionnaires to assess preferences for individual product traits, while this study brings a different approach by considering each characteristic as a part of a whole, including both physical and nutritional facts of the HEN.

The results of this survey depict the ideal HEN product, both for patients and physicians, as one that shows a good tolerability profile, is adaptable to patient’s comorbidities and has an adequate content of nutrients and calories. However, tolerability and nutritional facts were found to be more important for physicians than for patients. Handling easiness was found to be slightly important for both populations, while simplicity of connections was the least important HEN attribute, not even for patients.

Although the results found mainly meet expectations, a few aspects could be discussed. According to experts, the lack of importance of the information attribute could be explained by the fact that physicians usually get the information from other sources, while patients mainly trust in their prescriber. This same issue would justify the lower importance assigned by patients to HEN nutritional characteristics compared to physicians, as they are confident that they will make the best choice.

Conversely, it might seem strange that physicians gave lower RI to adaptability, but this is due to a group effect, i.e., physicians paid more attention to tolerability and nutritional facts, so, proportionally, adaptability was not so relevant.

Although groups were overall homogeneous, subgroup analyses showed that patients under 75 years were more concerned about “tolerability” than older ones (≥75 years). In contrast, container characteristics were more important for elderly patients. This might be related to the fact that elderly patients living at home had reported some handling difficulties, including opening or reading medication containers.

Finally, *ad hoc* questionnaire results showed that both groups of respondents mainly agreed upon the importance of these features, although physicians placed more importance on certain container characteristics such as “easy-handling bag”, “container material” or “reusable container” than patients, along with other factors relating to the “variety of flavors available” or the “duration of administration”. On the contrary, only the “allows oral and tube feeding” feature was more relevant for patients than for physicians.

These findings suggest that physicians are concerned about patients’ safety and quality of life, as they do not only value each of the different traits but they also pay special attention to administration duration (as it is usually related to tolerability issues) and HEN flavor (as it may affect gastric reflux).

A frequent source of uncertainty is the appropriateness of the attributes and selected levels, which may diverge depending on personal perceptions, and may even raise some skepticism about the results. However, the extensive literature review, followed by the validation carried out by the focus groups, might considerably reduce this uncertainty. Moreover, the significance of the results was high (except for information), confirming the adequateness of the choice made. However, as mentioned, this is the first Conjoint Analysis conducted to elicit patients’ and professionals’ preferences for HEN product characteristics, and further investigation might improve understanding.

Another frequently discussed issue is the representativeness of the participants. Even when they include a sufficiently large number of respondents, their overall defining traits can sometimes differ from those expected. However, in our sample of patients, the high proportion of patients with gastrostomy is remarkable. This is consistent with the fact that the inclusion criteria required them to have received HEN for at least 9 months, in this case gastrostomy being the recommended route of feeding. With respect to the underlying disease (50% neurological and 44% oncological), the proportion agreed with recently published data [9], according to which the most common primary diagnoses of HEN patients in Spain are neurological and oncological.

In conclusion, our findings show that although patients and physicians have a similar perception about the relevance of different HEN characteristics, physicians’ preferences are not completely in line with patients’ preferences. In this sense, our results could be helpful to further guide physicians to aggregate both points of view when selecting a HEN. This may lead to a more inclusive and patient-focused prescription, as well as generally improved adherence.

## Electronic supplementary material

Example of the choice set

Search terms and search strategy

Choice scenarios

Stepwise multilinear regression of relative importance (patients)

## References

[1] National Institute for Health and Care Excellence Guidance [Internet]. Nutrition support in adults: oral nutrition support, enteral tube feeding and parenteral nutrition. [Accessed: February 2006]. Available at: https://www.nice.org.uk/guidance/CG32. 31999417

[2] Ministerio de Sanidad y Consumo. Guía de nutrición enteral domiciliaria en el Sistema Nacional de Salud. [Accessed: June 2016] Available at: http://www.msssi.gob.es/profesionales/prestacionesSanitarias/publicaciones/docs/guiaNED.pdf.

[3] Cuerda C, Planas M, Gómez Candela C, Luengo LM, group NS (2009). Trends in home enteral nutrition in Spain: analysis of the NADYA registry 1992-2007. Nutr Hosp.

[4] Moreno Villares JM (2004). The practice of home artificial nutrition in Europe. Nutr Hosp.

[5] Van Gossum A (2005). Home enteral nutrition. Epidemiology and legislation in Europe. Nestle Nutr Workshop Ser Clin Perform Progr.

[6] Hebuterne X, Bozzetti F, Moreno Villares JM, Pertkiewicz M, Shaffer J, Staun M (2003). Home enteral nutrition in adults: a European multicentre survey. Clin Nutr.

[7] Delegge MH (2005). Home enteral nutrition. Demographics and utilization in the United States. Nestle Nutr Workshop Ser Clin Perform Progr.

[8] Olveira G, Tapia MJ, Colombo N, Muñoz A, Gonzalo M, C-Soriquer F (2009). Usefulness of the daily defined dose method to estimate trends in the consumption, costs and prevalence of the use of home enteral nutrition. Clin Nutr.

[9] Wanden-Berghe C, Luengo Pérez LM, Matía Martín P, Cuerda Compes C, Burgos Peláez R, Alvarez Hernández J (2014). Home enteral nutrition in Spain; NADYA registry 2011-2012. Nutr Hosp.

[10] Klek S, Hermanowicz A, Dziwiszek G, Matysiak K, Szczepanek K, Szybinski P (2014). Home enteral nutrition reduces complications, length of stay, and health care costs: results from a multicenter study. Am J Clin Nutr.

[11] Stratton RJ, Elia M (2008). British Artificial Nutrition Survey (BANS). A cost-utility analysis in patients receiving enteral tube feeding at home and in nursing homes. Proc Nutr Soc.

[12] Apezetxea A, Carrillo L, Casanueva F, Cuerda C, Cuesta F, Irles JA (2016). The NutriQoL® questionnaire for assessing health-related quality of life (HRQoL) in patients with home enteral nutrition (HEN): validation and first results. Nutr Hosp.

[13] Crosby J, Duerksen D (2005). A retrospective survey of tube-related complications in patients receiving long-term home enteral nutrition. Dig Dis Sci.

[14] Gómez Candela C, Cos Blanco A, García Luna PP, Pérez de la Cruz A, Luengo Pérez LM, Iglesias Rosado C (2003). Complications of enteral nutrition at home. Results of a multicentre trial. Nutr Hosp.

[15] Crosby J, Duerksen DR (2007). A prospective study of tube- and feeding-related complications in patients receiving long-term home enteral nutrition. JPEN J Parenter Enter Nutr.

[16] Kane S, Huo D, Aikens J (2003). Medication nonadherence and the outcomes of patients with quiescent ulcerative colitis. Am J Med.

[17] Hommel KA, Davis CM, Baldasano RN (2008). Medication adherence and quality of life in pediatric inflammatory bowel disease. J Pediatr Psychol.

[18] Kane S, Shaya F (2008). Medication non-adherence is associated with increased medical health care costs. Dig Dis Sci.

[19] Haynes RB. Determinants of compliance: The disease and the mechanics of treatment. Compliance in health care. Baltimore: Johns Hopkins University Press; 1979.

[20] Clark MD, Determann D, Petrou S, Moro D, de Bekker-Grob EW (2014). Discrete choice experiments in health economics: a review of the literature. Pharmacoeconomics.

[21] Bridges J, Kinter E, Kidane L (2008). Things are looking up since we started listening to patients: recent trends in the application of conjoint analysis in health 1970–2007. Patient.

[22] Ryan M, Gerard K (2003). Using discrete choice experiments to value health care programmes: current practice and future research reflections. Appl Health Econ Health Policy.

[23] Say RE, Thomson R (2003). The importance of patient preferences in treatment decisions-challenges for doctors. BMJ.

[24] Bridges JFP, Hauber AB, Marshall D, Lloyd A, Prosser LA, Regier DA (2011). Conjoint analysis applications in health: a checklist of the ISPOR good research practices for conjoint analysis task force. Value Health.

[25] Lancsar E, Louviere J (2008). Conducting discrete choice experiments to inform healthcare decision making: a user’s guide. Pharmacoeconomics.

[26] Johnson RF, Lancsar E, Marshall D, Kilambi V, Münhbacher A, Regier DA (2013). Constructing experimental design for discrete-choice experiments: report of the ISPOR conjoint analysis experimental design good research practices task force. Value Health.

[27] Skipper A, Bhac C, Gregoire MB (1999). Knowing brand name affects patient preferences for enteral supplements. J Am Diet Assoc.

[28] Wada S, Nakaji S, Umeda T, Takashi I, Oyama T, Chinda D (2004). Nutritional effects of supplementing liquid-formula diet with dietary fiber on elderly bed-ridden patients. Tohoku J Exp Med.

[29] Rahemtulla Z, Baldwin C, Spiro A, McCough C, Norman AR, Frost G (2005). The palatability of milk-based and non-milk-based nutritional supplements in gastrointestinal cancer and the effect of chemotherapy. Clin Nutr.

[30] Rubio MA, Arrieta JL, Ruiz M, Garrido J, Rubio JA, del Llano J (2008). Design and validation of a scale to assess preferences of type 2 diabetic patients towards different nutritional supplements. Nutr Hosp.

[31] Darmon P, Karsegard VL, Nardo P, Dupertuis YM, Pichard C (2008). Oral nutritional supplements and taste preferences: 545 days of clinical testing in malnourished in-patients. Clin Nutr.

[32] Aizaki H (2012). Basic functions for supporting an implementation of choice experiments in R. J Stat Softw.

[33] Cochran WG (1963). Sampling Techniques.

[34] Instituto Nacional de Estadística (INE). Estimaciones de la población actual de España a 1 de enero de 2013. Available at: http://www.ine.es/jaxi/menu.do?type=pcaxis&path=%2Ft20%2Fe260&file=inebase&L=0 [Accessed: February 2013].

[35] Soto A, Tofe S, Leon M, Garcia Luna PP (2003). Estudio sobre la situación organizativa y asistencial de la nutrición clinica hospitalaria en España: de 1195-2001. Endocrinol Nutr.

[36] Therneau TM. Package ‘survival’. Available at: https://cran.r-project.org/web/packages/survival/survival.pdf [Accessed: September 2015].

[37] R Core Team [Internet]. R: A language and environment for statistical computing. [Accessed: 2015 September] Available at:: www.R-project.org/ [Accessed: September 2015].

[38] Croissant Y [Internet]. Estimation of multinomial logit models in R: the mlogit packages. [Accessed: 2015 September] Available at: https://cran.r-project.org/web/packages/mlogit/mlogit.pdf [Accessed: September 2015].

[39] Guimaraes C, Marra CA, Gill S, Simpson S, Meneilly G, Queiroz RHC (2010). A discrete choice experiment evaluation of patient’s preferences for different risk, benefit and delivery attributes of insulin therapy for diabetes management. Patient Prefer Adherence.

[40] Brame R, Paternoster R, Mazerolle P, Piquero A (1998). Testing for the equality of Maximum likelihood regression coefficients between two independent equations. J Quant Criminol.

[41] Paternoster R, Brame R, Mazerolle P, Piquero A (1998). Using the correct statistical test for the equality of regression coefficients. Criminology.

[42] Martínez-Costa C, Calderón C, Gómez-López L, Borraz S, Pedrón-Giner C (2013). Satisfaction with gastrostomy feeding in caregivers of children with home enteral nutrition; application of the SAGA-8 questionnaire and analysis of involved factors. Nutr Hosp.

