# Supplementary material for: Patients’ and professionals’ preferences in terms of the attributes of home enteral nutrition products in Spain. A discrete choice experiment
Source: Eur J Clin Nutr. 2017 Dec 20;72(2):272–80. doi: 10.1038/s41430-017-0023-8 (PMC5842881; doi:10.1038/s41430-017-0023-8)
Supplement: Supplementary file 1 — Example of the choice set [file 41430_2017_23_MOESM1_ESM.docx]

***Figure S 1.*** *Example of the choice set*

| **Product A** | **Product B** |
| --- | --- |
| Easily tolerable | Easily tolerable. |
| Not adaptable to other comorbidities present | Not adaptable to other comorbidities present |
| Provides the nutrients and calories needed by the patient. | Does not provide the nutrients and calories needed by the patient |
| Its characteristics make package handling harder | Its characteristics make package handling easier |
| Product connections are easy to perform | Product connections are easy to perform |
| The container does not include information about the nutrient composition and branding | The container includes information about the nutrient composition and branding |
| Which product do you prefer?  I prefer A  I prefer B | |
