# Supplementary material for: Patients’ and professionals’ preferences in terms of the attributes of home enteral nutrition products in Spain. A discrete choice experiment
Source: Eur J Clin Nutr. 2017 Dec 20;72(2):272–80. doi: 10.1038/s41430-017-0023-8 (PMC5842881; doi:10.1038/s41430-017-0023-8)
Supplement: Supplementary file 2 — Search terms and search strategy [file 41430_2017_23_MOESM2_ESM.docx]

**Table S 1.** Search terms and search strategy

| **SEARCH TERMS** | |
| --- | --- |
| **Related to HEN** | |
| **#1** | Enteral nutrition |
| **#2** | Home enteral nutrition |
| **#3** | HEN |
| **Related to treatment** | |
| **#4** | Treatment |
| **#5** | Management |
| **#6** | Formula |
| **Related to preference studies** | |
| **#7** | Conjoint analysis |
| **#8** | Choice model |
| **#9** | Stated preference |
| **#10** | Discrete choice |
| **#11** | DCE |
| **#12** | Trade-off |
| **#13** | Preference |
| **#14** | Willingness to pay |
| **#15** | Willingness to accept |
| **#16** | WTP |
