# Supplementary material for: Patients’ and professionals’ preferences in terms of the attributes of home enteral nutrition products in Spain. A discrete choice experiment
Source: Eur J Clin Nutr. 2017 Dec 20;72(2):272–80. doi: 10.1038/s41430-017-0023-8 (PMC5842881; doi:10.1038/s41430-017-0023-8)
Supplement: Supplementary file 3 — Choice scenarios [file 41430_2017_23_MOESM3_ESM.docx]

**Table S 2.** Choice scenarios

| Scenario | Tolerability | Adaptation | Nutrients | Handling | Connexions | Information |
| --- | --- | --- | --- | --- | --- | --- |
| 1 | Easy | Does not adapt | Intake | Difficult | Easy | Does not include |
|  | Easy | Does not adapt | No intake | Easy | Easy | Includes |
| 2 | Difficult | Adapts | Intake | Easy | Easy | Includes |
|  | Easy | Does not adapt | Intake | Difficult | Difficult | Includes |
| 3 | Easy | Does not adapt | Intake | Easy | Difficult | Includes |
|  | Difficult | Adapts | Intake | Difficult | Easy | Includes |
| 4 | Difficult | Adapts | No intake | Easy | Easy | Does not include |
|  | Difficult | Adapts | No intake | Easy | Difficult | Includes |
| 5 | Easy | Does not adapt | No intake | Easy | Difficult | Does not include |
|  | Difficult | Does not adapt | No intake | Difficult | Difficult | Does not include |
| 6 | Difficult | Does not adapt | No intake | Difficult | Easy | Includes |
|  | Difficult | Does not adapt | Intake | Easy | Easy | Does not include |
| 7 | Difficult | Adapts | Intake | Difficult | Difficult | Does not include |
|  | Easy | Adapts | No intake | Difficult | Easy | Does not include |
| 8 | Easy | Adapts | No intake | Difficult | Difficult | Includes |
|  | Easy | Adapts | Intake | Easy | Difficult | Does not include |
