# Supplementary material for: Patients’ and professionals’ preferences in terms of the attributes of home enteral nutrition products in Spain. A discrete choice experiment
Source: Eur J Clin Nutr. 2017 Dec 20;72(2):272–80. doi: 10.1038/s41430-017-0023-8 (PMC5842881; doi:10.1038/s41430-017-0023-8)
Supplement: Supplementary file 4 — Stepwise multilinear regression of relative importance (patients) [file 41430_2017_23_MOESM4_ESM.docx]

**Table S 3**. Stepwise multilinear regression of relative importance (patients)

| *Attribute* | *Coefficient* | *SE* | *p-value* |
| --- | --- | --- | --- |
| **Hospital location** | | | |
| Hardly tolerable | -2.223 | 0.239 | <0.001 |
| Not adaptable | -2.272 | 0.271 | <0.001 |
| Does not provide nutrients and calories | -1.612 | 0.130 | <0.001 |
| Hard to handle | -0.561 | 0.113 | 0.000 |
| Difficult connexions | 0.030 | 0.164 | 0.856 |
| *Madrid* | -0.805 | 0.371 | 0.030 |
| *Ciudad Real* | -0.325 | 0.252 | 0.197 |
| *Murcia* | -0.292 | 0.255 | 0.252 |
| *Barcelona* | -0.341 | 0.267 | 0.202 |
| *A Coruña* | -0.256 | 0.232 | 0.270 |
| **Need caregivers to answer on their behalf** | | | |
| Hardly tolerable | -2.219 | 0.239 | <0.001 |
| Not adaptable | -2.269 | 0.270 | <0.001 |
| Does not provide nutrients and calories | -1.608 | 0.129 | <0.001 |
| Hard to handle | -0.560 | 0.113 | <0.001 |
| Difficult connexions | 0.030 | 0.163 | 0.852 |
| *Need caregiver to answer* | -0.303 | 0.156 | 0.052 |
| **Age** | | | |
| Hardly tolerable | -2.253 | 0.241 | <0.001 |
| Not adaptable | -2.265 | 0.271 | <0.001 |
| Does not provide nutrients and calories | -1.618 | 0.130 | <0.001 |
| Hard to handle | -0.546 | 0.113 | 0.000 |
| Difficult connexions | 0.040 | 0.164 | 0.809 |
| ***Over 75 years*** | **0.376** | **0.177** | **0.033** |
| **Gender** | | | |
| Hardly tolerable | -2.212 | 0.239 | <0.001 |
| Not adaptable | -2.262 | 0.270 | <0.001 |
| Does not provide nutrients and calories | -1.602 | 0.129 | <0.001 |
| Hard to handle | -0.559 | 0.113 | <0.001 |
| Difficult connexions | 0.031 | 0.163 | 0.849 |
| *Female* | 0.112 | 0.160 | 0.484 |
| **Need of caregiver** | | | |
| Hardly tolerable | -2.211 | 0.238 | <0.001 |
| Not adaptable | -2.261 | 0.270 | <0.001 |
| Does not provide nutrients and calories | -1.602 | 0.129 | <0.001 |
| Hard to handle | -0.559 | 0.113 | <0.001 |
| Difficult connexions | 0.031 | 0.163 | 0.849 |
| *Need caregiver* | -0.069 | 0.195 | 0.724 |
| **Associated disease** | | | |
| Hardly tolerable | -2.226 | 0.239 | <0.001 |
| Not adaptable | -2.274 | 0.271 | <0.001 |
| Does not provide nutrients and calories | -1.616 | 0.130 | <0.001 |
| Hard to handle | -0.561 | 0.113 | <0.001 |
| Difficult connexions | 0.029 | 0.164 | 0.862 |
| *Neuropathy* | -0.078 | 0.249 | 0.755 |
| *H&N cancer* | -0.153 | 0.230 | 0.505 |
| *GI cancer* | -0.663 | 0.304 | 0.029 |
| *Dementia* | 0.047 | 0.308 | 0.878 |
| *GI disease* | -0.489 | 0.457 | 0.285 |
| *Other cancers* | 0.170 | 0.586 | 0.771 |
| *Others* | 0.093 | 0.514 | 0.856 |
| **HEN type** | | | |
| Hardly tolerable | -2.211 | 0.238 | <0.001 |
| Not adaptable | -2.261 | 0.270 | <0.001 |
| Does not provide nutrients and calories | -1.601 | 0.129 | <0.001 |
| Hard to handle | -0.559 | 0.113 | <0.001 |
| Difficult connexions | 0.031 | 0.163 | 0.849 |
| *Nutritional support* | 0.064 | 0.238 | 0.790 |
| **Route of administration** | | | |
| Hardly tolerable | -2.211 | 0.238 | <0.001 |
| Not adaptable | -2.261 | 0.270 | <0.001 |
| Does not provide nutrients and calories | -1.602 | 0.129 | <0.001 |
| Hard to handle | -0.559 | 0.113 | <0.001 |
| Difficult connexions | 0.031 | 0.163 | 0.849 |
| *Gastrostomy* | -0.030 | 0.174 | 0.862 |
| *Jejunostomy* | -0.169 | 0.411 | 0.680 |
| **Method of administration** | | | |
| Hardly tolerable | -2.197 | 0.239 | <0.001 |
| Not adaptable | -2.255 | 0.271 | <0.001 |
| Does not provide nutrients and calories | -1.602 | 0.130 | <0.001 |
| Hard to handle | -0.563 | 0.113 | <0.001 |
| Difficult connexions | 0.021 | 0.164 | 0.900 |
| *Gravity* | 0.210 | 0.178 | 0.240 |
| *Infusion pump* | -0.254 | 0.270 | 0.346 |
